# Is rs759853 polymorphism in promoter of aldose reductase gene a risk factor for diabetic nephropathy? A meta-analysis

**DOI:** 10.1186/s40001-015-0089-5

**Published:** 2015-02-10

**Authors:** Wenpeng Cui, Bing Du, Yingchun Cui, Lili Kong, Hao Wu, Yangwei Wang, Lining Miao, Wenhua Zhou

**Affiliations:** Department of Nephrology, Second Hospital of Jilin University, 218 Ziqiang Street, Changchun, Jilin 130041 China; Department of Cardiology, the Second Part of First Hospital, Jilin University, 3302 Jilin Road, Changchun, Jilin 130032 China

**Keywords:** Aldose reductase, Diabetic nephropathy, Meta-analysis

## Abstract

**Background:**

So far, a number of case-control or cohort studies have been carried out to investigate the relationship between rs759853 polymorphism in the promoter of aldose reductase (AR) gene and the risk of diabetic nephropathy (DN). However, the results have generated considerable controversy. We performed this study to clarify the linkage between this gene mutation and the risk of DN.

**Methods:**

A comprehensive literature search of electronic databases and a well-organized meta-analysis were conducted.

**Results:**

Twelve comparisons and 4,735 individuals from nine published case-control or cohort studies were included finally. From none to large heterogeneity was observed, therefore, both fixed and random models were used. Significant differences were found between AR rs759853 polymorphism and susceptibility of DN from both type 1 and type 2 diabetes in all genetic models (allele contrast, OR = 1.37, CI (1.18, 1.59), *P* < 0.0001; additive model, OR = 1.78, CI (1.25, 2.53), *P* = 0.01; recessive model OR = 1.33 CI (1.08, 1.63), *P* = 0.008; dominant model, OR = 1.52, CI (1.26, 1.84), *P* < 0.0001; codominance model OR = 1.30 (1.15, 1.47), *P* < 0.0001). In stratified meta-analyses for type 2 diabetes by ethnicity, the significant relationship was found in allele contrast and dominant model in Caucasians, and in allele contrast and codominance model in Asians. However, data do not support the linkage between this gene mutation and the progression of DN. There was no significant publication bias.

**Conclusions:**

The evidence currently available shows that the AR rs759853 polymorphism may correlate with the susceptibility of DN. However, data do not support the association between this DNA variation and the progression of DN.

## Background

Diabetic nephropathy (DN), a serious chronic microvascular complication of both type 1 and type 2 diabetes mellitus, is the leading cause of end-stage renal disease worldwide [1]. In 2009, the cost of dialysis treatment of diabetes induced end-stage renal disease patients accounted for approximately 17 billion dollars in the United States. Despite lots of clinical and basic studies performed, the pathogenesis and mechanism of DN have not been definitively explained. Only one third of subjects with diabetes develop DN, indicating that only a group of specific diabetes patients are at risk. Although hyperglycemia is one of the most important risk factors for the development of DN, accumulating evidences indicate that genetic susceptibility has also been proposed to contribute to DN susceptibility and occurrence [2].

As a member of the aldo-keto reductase superfamily, aldose reductase (AR) is the first rate-limiting enzyme of the polyol pathway and catalyses NADPH-dependent reduction of glucose to sorbitol [3]. This pathway is generally active under hyperglycemia conditions only, leading to accumulation of intracellular sorbitol [4]. Sorbitol may cause tissue damage by leading to hyperosmotic stress, because it cannot easily pass through the cell membranes [5]. Excessive accumulation of intracellular sorbitol has been observed in both renal glomeruli of streptozotocin-induced diabetic rats [6] and mesangial cells stimulated by high concentration of glucose [7]. In addition, AR gene expression and enzyme activity were declared to be increased in mouse podocytes under high-glucose condition [8]. Furthermore, for clinical studies, overexpression of AR in protein level can also be found in diabetic patients [9,10]. Finally, administration of AR inhibitor may prevent the process of incipient DN in patients with type 2 diabetes [11]. These findings support the hypothesis that AR gene upregulation may be one of the multiple risk factors of developing DN.

C-106 T single nucleotide polymorphism (rs759853) is a mutation of C to T at nucleotide 106 in the promoter of AR gene. This mutation has been well studied in a growing body of studies which examined the association between rs759853 polymorphism and the risk of DN in several populations. However, these results have generated considerable controversy [12-20]. For DN in type 2 diabetes, rs759853 polymorphism was confirmed either to be a risk [12,15-17,21] or a neutral [13,14] factor for the development of this disease. Similarly, discrepant conclusions were also shown in researches conducted on type 1 diabetic subjects, apparent relationship between rs759853 polymorphism in AR gene and susceptibility of DN was revealed in both British population and American population [19,20] but not shown in a French investigation [18]. Therefore, a meta-analysis was performed to evaluate the overall evidences of all available studies about rs759853 polymorphism in AR gene and the risk of DN.

## Methods

### Search strategy

To identify eligible literatures, we conducted a comprehensive literature search of electronic Embase, PubMed, and OVID databases. MeSH terms such as ‘diabetic nephropathy’, ‘aldose reductase’, ‘gene polymorphism’, and ‘single nucleotide polymorphism’ were used to obtain studies published from 1983 to 2013 (last search updated on 31 December 2013). We also reviewed all the references cited in these articles to identify additional relevant publications.

### Selection criteria

Selection criteria were established before literature searching to avoid selection bias. Abstracts, editorials, case reports, and review articles were excluded. Studies included in this meta-analysis should meet all of the following criteria: (1) an unrelated case-control or perspective cohort design was used, (2) allele frequency and genotype distribution were available or could be calculated, (3) rs759853 polymorphism in AR gene in group diabetes was in Hardy-Weinberg equilibrium (HWE), and (4) all cases should be diagnosed as DN and the controls were diabetes patients without DN. Two authors identified articles eligible for further review by performing an initial screen of the abstracts or titles of the search results. The second screening was based on a full-text review according to the selection criteria. Discrepancies were resolved by discussion and consultation with other authors.

### Data extraction

Information was carefully extracted from all eligible studies independently by two authors. Different viewpoints were resolved by discussion between the two. The following information was extracted from each study: name of first author, year of publication, racial decent, diagnostic standard of diabetes and DN, study design, type of diabetes, genotyping method, clinical characteristics (age, duration of diabetes, and gender), HWE *P* value, sample size, genotype distribution, and allele frequency. In some studies HWE *P* value, allele frequency and/or genotype distribution were not provided directly, we calculated them from the data provided using standard formulae.

### Statistical analysis

This meta-analysis examined the overall association with increased risk of DN between allele T and allele C. The relationship was also tested in additive model (TT vs CC), recessive model (TT vs TC + CC), dominant model (TT + TC vs CC), and with codominance model (TC vs TT + CC). Review Manager 5.0, one of the statistical software packages for managing and analyzing all aspects of a Cochrane Collaboration systematic review, was used in current study. The effect of association was indicated as odds ratio (OR) with the corresponding 95% confidence interval (CI). The OR was defined as the odds of developing DN in those who carried this rs759853 polymorphism compared with the odds in those who did not carry it. The ORs of different comparisons were combined by using the random-effects model of DerSimonian and Laird if true between-study heterogeneity exist or else by using Mantel and Haenszel fixed-effects model instead [22]. We used the Q-statistic which is traditionally considered statistically significant for *P* < 0.10, to test heterogeneity between studies [23]. Heterogeneity was also quantified with the *I*^2^ metric, which is independent of the number of studies included in meta-analysis [24]. The *I*^2^ takes values between 0% and 100%, with higher values denoting greater degree of heterogeneity (*I*^2^ = 0% to 25%, none heterogeneity; *I*^2^ = 25% to 50%, moderate heterogeneity; *I*^2^ = 50% to 75%, large heterogeneity; *I*^2^ = 75% to 100%, extreme heterogeneity). HWE of AR rs759853 polymorphism in group diabetes was detected by the chi-square test. Visual analysis of the funnel plot and Egger’s linear regression test by using Stata statistical software (version 12.0) were used to assess the publication bias. The significance of the intercept was determined by *t*-test, and *P* < 0.05 was considered statistically significant.

## Results

### Literature retrieval

**Figure 1:**
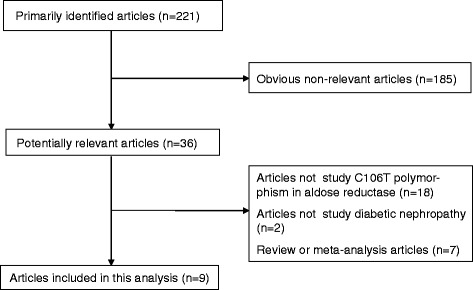
**Flow chart of article selection approach.**

The combined search yielded at least 221 references. After discarding those that did not meet the criteria, nine relevant case-control or prospective cohort studies were included [12-21]. Of these nine studies, one study contained subjects from four subgroups [19], so a total of 12 separate comparisons were considered. A diagram flow summarizing the process of study selection is shown in Figure 1. In addition, racial decent, diagnostic standard of DN and diabetes, study design, type of diabetes, clinical characteristics (age, duration of diabetes, and gender), HWE *P* value, and sample size of each study are listed in Table 1.

**Table 1:** **Characteristics and summary data of the included studies**

**Studies (first author publication years)**	**Racial decent (country)**	**Disease diagnostic standard**	**Study design**	**Type of diabetes**	**Clinical characteristics (control/case)**			**HWE** ***P*** **value**	**Sample size (control/case)**	**Genotype distribution (control/case)**			**Frequency of allele T (%) (control/case)**
					**Age (year)**	**Duration of diabetes (year)**	**Male (%)**			**T/T**	**T/C**	**C/C**	
So et al. [12]	Asians (China)	Diabetes: not mentioned; DN: GFR < 60 ml/min/1.72 m^2^	Prospective design	Type 2	-/-	7.5/8.0	-/-	>0.05	866/208	44/13	269/76	553/119	18.6/22.2
Wolford et al. [13]	Caucasians (America)	Diabetes: not mentioned; DN: ACR ≥ 300 mg/g	Case-control design	Type 2	55.9/58.0	20.4/20.7	43.9/31.5	>0.05	105/103	5/1	26/32	74/70	17.1/16.5
Gosek et al. [14]	Caucasians (Poland)	Diabetes: NDDG (1979); DN: ACR ≥ 1.9 mg/mmol (men) or ≥2.8 mg/mmol (women)	Prospective design	Type 2	-/-	14.0/-	-/-	>0.05	162/282	20/40	85/145	57/97	38.6/39.9
Sivenius et al. [15]	Caucasians (Finland)	Diabetes: WHO (1980); DN: UAE > 30 mg/24 h	Prospective design	Type 2	-/-	10.0/10.0	-/-	>0.05	68/17	1/2	17/7	50/8	14.6/32.4
Makiishi et al. [16]	Asians (Japan)	Diabetes: WHO (1999); DN: AER ≥ 20 μg/min	Case-control design	Type 2	65.0/64.0	19.0/17.0	46.5/55.6	>0.05	228/230	3/13	66/63	159/144	15.8/20.2
Wang et al. [17]	Asians (China)	Diabetes: not mentioned; DN: AER ≥ 20 μg/min	Case-control design	Type 2	52.3/60.8	4.2/8.1	38.9/46.1	>0.05	458/280	26/15	130/96	302/169	19.9/22.5
Fanelli et al. [18]	Caucasians (France)	Diabetes: not mentioned; DN: not mentioned	Prospective design	Type 1	-/-	14.6/22.2	-/-	>0.05	157/335	33/61	69/161	55/113	44.8/42.2
Neamat-Allah et al. [19]	Caucasians (Ireland)	Diabetes: not mentioned DN: UAE > 300 mg/24 h	Case-control design	Type 1	-/-	-/-	-/-	>0.05	102/107	7/22	31/44	64/41	22.1/40.0
Neamat-Allah et al. [19]	Caucasians (England)	Diabetes: not mentioned DN: UAE > 300 mg/24 h	Case-control design	Type 1	-/-	29.0/26.3	48.9/61.2	>0.05	85/77	12/19	30/39	43/19	31.8/50.0
Neamat-Allah et al. [19]	Caucasians (England)	Diabetes: not mentioned DN: UAE > 300 mg/24 h	Case-control design	Type 2	-/-	8.9/12.6	54.4/65.9	>0.05	146/85	33/20	48/45	65/20	39.0/50.0
Neamat-Allah et al. [19]	Pima Indians (America)	Diabetes: not mentioned DN: UAE > 300 mg/24 h	Case-control design	Type 2	-/-	-/-	-/-	>0.05	154/181	4/7	40/61	110/113	15.6/20.7
Moczulski et al. [20]	Caucasians (America)	Diabetes: not mentioned DN: ACR ≥ 17 μg /mg (men) or ≥25 μg /mg (women)	Case-control design	Type 1	35.5/36.3	23.3/24.9	-/-	>0.05	193/221	22/35	83/112	88/74	32.9/41.2

*Abbreviations:* *ACR* albumin-creatinine ratio, *AER* albumin excretion rate, *DN* diabetic nephropathy, *GFR* glomerular filtration rate, *HWE* Hardy-Weinberg equilibrium, *NDDG* National Diabetes Data Group, *UAE* urinary albumin excretion.

### Statistics summary

Overall, studies included in our meta-analysis provided 2,724 controls (diabetes) and 2,011 cases (DN). All studies used polymerase chain reaction-restriction fragment length polymorphism to detect genotype. The allele T prevalence of rs759853 polymorphism in groups diabetes and DN was 23.5% and 31.9%, respectively. TT prevalence in diabetes group and DN group was 7.7% and 11.6%, respectively, and the proportion of CC among diabetes group and DN group was 59.5% and 50.0%, respectively. The genotype and allele frequencies of the individual studies are listed in Table 1.

### Susceptibility of DN

**Figure 2:**
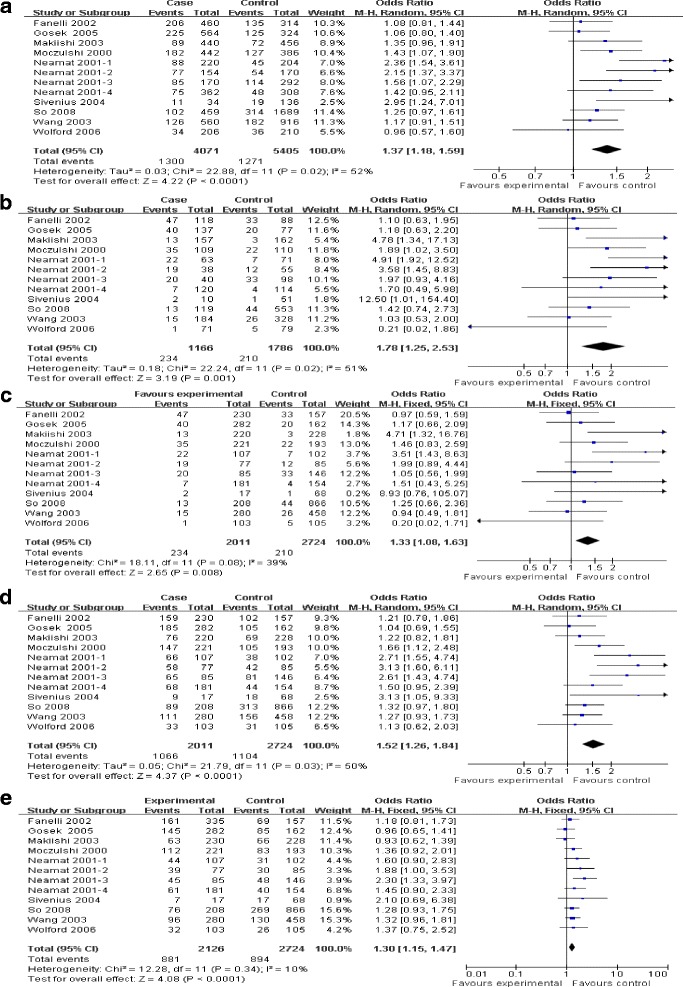
**Main results of the association between rs759853 polymorphism in AR gene and the susceptibility of DN.** Each study is shown by a point estimate of the OR and the accompanying 95% CI. **(a)** Allele contrast, **(b)** additive model, **(c)** recessive model, **(d)** dominant model, and **(e)** codominance model. Abbreviations: AR, aldose reductase; CI, confidence interval; DN, diabetic nephropathy; OR, odds ratio.

**Figure 3:**
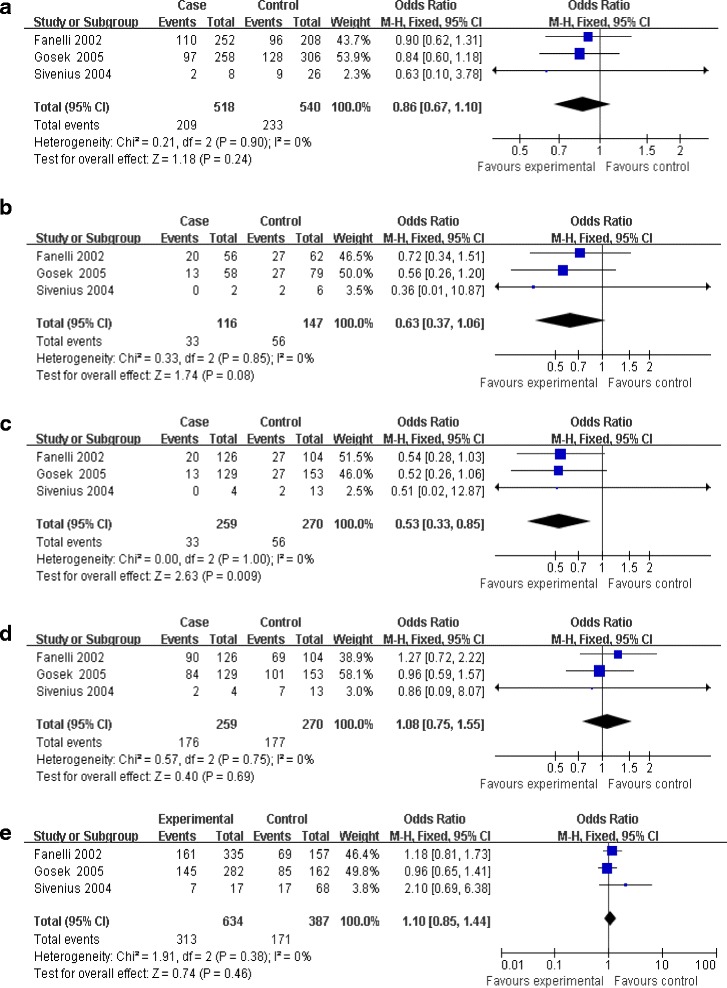
**Main results of the association between rs759853 polymorphism in AR gene and the progression of DN.** Each study is shown by a point estimate of the OR and the accompanying 95% CI. **(a)** Allele contrast, **(b)** additive model, **(c)** recessive model, **(d)** dominant model, and **(e)** codominance model. Abbreviations: AR, aldose reductase; CI, confidence interval; DN, diabetic nephropathy; OR, odds ratio.

All included 12 comparisons demonstrated the association between AR rs759853 polymorphism and the susceptibility of DN. Main analyses for investigating the association between rs759853 allele T and the susceptibility of DN relative to allele C revealed large heterogeneity and random effects pooled OR was significant (OR = 1.37, CI (1.18, 1.59), *P* < 0.0001). Likewise, obviously significant results were also found in additive model, recessive model, dominant model, and codominance model for allele T (additive model, OR = 1.78, CI (1.25, 2.53), *P* = 0.01; recessive model, OR = 1.33 CI (1.08, 1.63), *P* = 0.008; dominant model, OR = 1.52, CI (1.26, 1.84), *P* < 0.0001; codominance model, OR = 1.30 (1.15, 1.47), *P* < 0.0001) (Table 2, Figures 2 and 3).

**Table 2:** **ORs with CIs and heterogeneity results for the various allele/genotype contrasts**

	**Populations**	**Studies (** ***n*** **)**	**Allele/genotypes (n)**	***I*** ^***2***^ **(%)**	***P*** **1**	**OR (95% CI)**	***P*** **2**
Allele contrast	Susceptibility	12	9,476	52	0.02	Random: 1.37 (1.18, 1.59)	<0.0001
	Type 1	4	2,350	35	0.20	Fixed: 1.75 (1.48, 2.07)	<0.00001
	Type 2	8	7,126	16	0.31	Fixed: 1.25 (1.11, 1.41)	0.0003
	Caucasians	5	2,606	49	0.10	Fixed: 1.26 (1.05, 1.50)	0.01
	Asians	3	4,520	0	0.80	Fixed: 1.24 (1.06, 1.45)	0.008
	Progression	3	1,058	0	0.90	Fixed: 0.86 (0.67, 1.10)	0.24
	Type 2	2	598	0	0.76	Fixed: 0.83 (0.59, 1.16)	0.27
Additive model	Susceptibility	12	3,009	51	0.02	Random: 1.78 (1.25, 2.53)	0.001
	Type 1	4	652	68	0.02	Random: 2.27 (1.19, 4.33)	0.01
	Type 2	8	2,357	38	0.12	Fixed: 1.44 (1.07, 1.95)	0.02
	Caucasians	5	797	43	0.13	Fixed: 1.40 (0.92, 2.13)	0.12
	Asians	3	1,560	55	0.11	Random: 1.60 (0.80, 3.20)	0.19
	Progression	3	263	0	0.85	Fixed: 0.63 (0.37, 1.06)	0.08
	Type 2	2	145	0	0.81	Fixed: 0.54 (0.26, 1.15)	0.11
Recessive model	Susceptibility	12	4,735	39	0.08	Fixed: 1.33 (1.08, 1.63)	0.008
	Type 1	4	1,172	56	0.08	Random: 1.47 (1.08, 2.02)	0.02
	Type 2	8	3,563	34	0.16	Fixed: 1.22 (0.92, 1.61)	0.17
	Caucasians	5	1,303	27	0.24	Fixed: 1.12 (0.76, 1.64)	0.58
	Asians	3	2,260	60	0.08	Random: 1.47 (0.71, 3.05)	0.30
	Progression	3	529	0	1.00	Fixed: 0.53 (0.33, 0.85)	0.009
	Type 2	2	299	0	0.99	Fixed: 0.52 (0.26, 1.04)	0.07
Dominant model	Susceptibility	12	4,735	50	0.03	Random: 1.52 (1.26, 1.84)	<0.0001
	Type 1	4	1,172	63	0.04	Random: 1.93 (1.28, 2.91)	0.002
	Type 2	8	3,563	27	0.22	Fixed: 1.34 (1.15, 1.55)	0.0001
	Caucasians	5	1,303	55	0.06	Fixed: 1.45 (0.99, 2.12)	0.06
	Asians	3	2,260	0	0.95	Fixed: 1.28 (1.06, 1.55)	0.01
	Progression	3	529	0	0.75	Fixed: 1.08 (0.75, 1.55)	0.69
	Type 2	2	299	0	0.92	Fixed: 0.96 (0.55, 1.55)	0.85
Codominance model	Susceptibility	12	4,735	10	0.34	Fixed: 1.30 (1.15, 1.47)	<0.0001
	Type 1	4	1,172	0	0.61	Fixed: 1.38 (1.10, 1.74)	0.005
	Type 2	8	3,563	30	0.19	Fixed: 1.26 (1.09, 1.47)	0.002
	Caucasians	5	1,303	45	0.12	Fixed: 1.36 (1.08, 1.72)	0.01
	Asians	3	2,260	1	0.36	Fixed: 1.20 (0.98, 1.46)	0.07
	Progression	3	529	0	0.38	Fixed: 1.10 (0.85, 1.44)	0.46
	Type 2	2	299	44	0.19	Fixed: 1.04 (0.72, 1.50)	0.83

*Abbreviations:* *AR* aldose reductase, *CI* confidence interval, *DN* diabetic nephropathy, *OR* odds ratio, *P1P* value for heterogeneity, *P2P* value for significance with fixed or random effects model.

Additionally, subgroup analyses for DN in type 1 diabetic individuals yielded a significant relationship between this gene polymorphism and the susceptibility of DN in all genetic models. For patients with DN derived from type 2 diabetes, positive results were produced in allele contrast, additive model, dominant model, together with codominance model. What is more, further stratified analyses for DN in type 2 diabetes individuals by ethnicity exhibited a positive correlation with the increased risk of DN among Caucasians population both in allele contrast and codominance model. Significant results in allele contrast and dominance model were found in Asians individuals (Table 2).

### Progression of DN

Three investigations delineated the difference of AR rs759853 polymorphism between micro- and macro-proteinuria [14,15,18]. No heterogeneity existed between the three comparisons, thus fixed model was used in subsequent analyses. Significant results were found only in recessive model (OR = 0.53, CI (0.33, 0.85), *P* = 0.009) but not in allele contrast, additive model, dominant model, or codominance model (allele contrast, OR = 0.86, CI (0.67, 1.10), *P* = 0.24; additive model, OR = 0.63, CI (0.37, 1.06), *P* = 0.08; dominant model, OR = 1.08, CI (0.75 1.55), *P* = 0.69; codominance model, OR = 1.10, CI (0.85, 1.44), *P* = 0.46) (Table 2, Figures 2 and 3).

Furthermore, subgroup analyses for DN in type 2 diabetes revealed that no significant difference was observed in all genetic models for main analyses (Table 2).

### Publication bias

**Figure 4:**
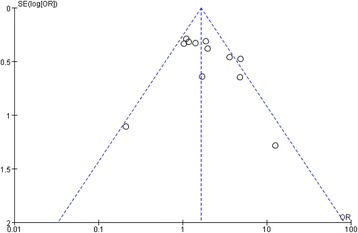
**Funnel plot of publication bias.**

Probability of the existing of publication bias was low because the funnel plot was symmetrical based on a visual analysis (Figure 4). We further used Egger’s linear regression to evaluate the publication bias. The results of Egger’s test showed there was no publication bias for the main analysis for DN susceptibility in additive model (*P* = 0.31).

## Discussion

Association study based on the comparison of genotype and allele distribution in cases and controls is considered a useful approach to investigate the role of candidate genes in the development and progression of multifactorial diseases [25,26]. In the current meta-analysis, the effects of allele contrast, together with additive, recessive, dominant, and codominance models, were estimated. The results of the main analyses demonstrated definite relationship between AR rs759853 polymorphism and the susceptibility of DN in all genetic models. Our results were consistent with those of previous studies [12,15-17,19,20]; however, these pooled results should be considered cautiously. Age, duration of diabetes, and gender are independent risk factors which are associated with increased rates of type 2 diabetes. These factors should be well matched between cases and controls, while such information was not available in some of published literatures [12,14,15]. Furthermore, heterogeneity in genetic models fluctuated from none to large, which might decrease the effects of pooled results.

Since there were four and eight comparisons studying type 1 and type 2 diabetes in the present meta-analysis, respectively, subgroup analyses by the type of diabetes were performed. Significant associations between AR rs759853 polymorphism and risk of DN in both types of diabetic individuals were also found in all genetic models, except for subgroup analysis for type 2 diabetes in recessive model. Noteworthily, from none to large heterogeneity was still exit when subgroup analyses were performed. As we know, one of the most important factors cause heterogeneity between studies is population diversity. Therefore, according to previous studies [27,28] and comparisons included in our meta-analysis, we further categorized the type 2 diabetes participants by ethnicity into three groups: Caucasians, Asians, and ‘non-Caucasians, non-Asians.’ For both Caucasians and Asians, we succeeded in finding positive relationship between this DNA sequence variation and susceptibility of DN. Because there was only one comparison in ‘non-Caucasians, non-Asians’ group (Pima Indians) [19], we did not perform subgroup analysis for this group.

The exact molecular mechanism of the observed association between rs759853 polymorphism in AR gene and susceptibility of DN is still unclear. One possible situation might be this polymorphism is located in the promoter of AR gene, which might affect the transcription of this gene. This hypothesis was supported by a previous Japanese study [16]. Makiishi et al. found that erythrocyte AR content in subjects with TT genotype was significantly greater than that in subjects with the TC and CC genotypes; furthermore, erythrocyte AR content in DN patients was also significantly greater than that in type 2 diabetes patients [16]. However, conflicting report claimed that allele C has a much higher transcriptional activity of the AR gene *in vitro* compared with allele T [29]. One explanation for the discrepancy between these two studies is only haplotypes of (AC) n repeats and C (106) T polymorphisms have been studied in this *in vitro* experiment by Yang et al [29]. Therefore, direct evidence was lack because the transcriptional effect comparison between allele C and allele T of single nucleotide polymorphism (SNP) at nucleotide 106 in the promoter of AR gene was not shown. Additionally, as we all know, the situations between *in vivo* and *in vitro* studies are quite different, therefore, more studies focusing on this issue are needed.

In addition, three comparisons revealed the effects of this DNA sequence mutation in AR gene on the progression of DN [14,15,18]. Generally, no obvious evidences were found in either main analyses or subgroup analyses for type 2 diabetes about the association between AR rs759853 polymorphism and the disease cause of DN, though subgroup analyses revealed significant result in recessive model. This result was difficult for us to explain, one of the reason may be the lack of comparisons and small sample size. Therefore, more evidence is needed to test and reconfirm this hypothesis in future.

Recent advances have made it possible to genotype the human genome at up to a million polymorphic sites in thousands of samples within a reasonable time frame. This has heralded the era of genome-wide association studies (GWASs) which have emerged as powerful tools for the dissection of complex genetic traits, such as susceptibility to DN [30,31]. This technology facilitated rapid progress in DN genetic research. Though we failed to find directed evidences about the association between AR rs759853 polymorphism and DN from previous GWASs, it is possible that some of the specific SNP candidates are in high linkage disequilibrium with rs759853 polymorphism in AR gene.

In a previous meta-analysis [19], the authors also provided the conclusion that rs759853 polymorphism positively correlated with the development of DN from both type 1 and type 2 diabetes based on two articles [19,20]. Compared to the study by Neamat-Allah et al. [19], our meta-analysis has some advantages. First, we updated this analysis by adding another seven studies, which enlarged the sample size and made the results more stable. Second, we made subgroup analyses by ethnicity, which provided data for individual population. Third, we also explored whether rs759853 polymorphism influence the disease process of DN. What is more, all studies included in our meta-analysis were in HWE, which may increase the stability of results.

Despite these advantages, as for all meta-analysis, the results of the present study should be considered cautiously. First, some articles did not provide necessary clinical information, such as age, duration of diabetes, and gender, which made us unable to estimate whether the participants in controls were well matched with that in cases. Second, from moderate to large significant of heterogeneity was found when investigating the relationship between AR rs759853 polymorphism and the susceptibility of DN, which suggested that analytic results should be considered cautiously.

## Conclusions

Overall, the evidence currently available shows significant correlations between AR rs759853 polymorphism and the susceptibility to DN from both types of diabetes. In stratified meta-analyses for type 2 diabetes, similar results were found in both Caucasians and Asians. However, data do not support the linkage between this gene mutation and the progression of DN. Taking into account those limitations of this meta-analysis, additional studies with more strict selection of patients, much larger sample size, and well-matched controls will be needed. Moreover, gene-gene and gene-environment interactions should also be considered in future studies.

## Abbreviations

AR: Aldose reductase
CI: Confidence interval
DN: Diabetic nephropathy
GWASs: Genome-wide association studies
HWE: Hardy-Weinberg equilibrium
OR: Odds ratio
SNP: Single nucleotide polymorphism

## References

[1] Hasslacher C, Ritz E, Wahl P, Michael C (1989). Similar risks of nephropathy in patients with type I or type II diabetes mellitus. Nephrol Dial Transplant..

[2] Du B, Liu S, Cui C, Wang S, Cui W (2013). Association between glucose transporter 1 rs841853 polymorphism and type 2 diabetes mellitus risk may be population specific. J Diabetes.

[3] Wang K, Bohren KM, Gabbay KH (1993). Characterization of the human aldose reductase gene promoter. J Biol Chem..

[4] Greene DA, Lattimer SA, Sima AA (1987). Sorbitol, phosphoinositides, and sodium-potassium-ATPase in the pathogenesis of diabetic complications. N Engl J Med..

[5] Thomas TP, Porcellati F, Kato K, Stevens MJ, Sherman WR, Greene DA (1994). Effects of glucose on sorbitol pathway activation, cellular redox, and metabolism of myo-inositol, phosphoinositide, and diacylglycerol in cultured human retinal pigment epithelial cells. J Clin Invest..

[6] Cohen MP (1986). Aldose reductase, glomerular metabolism, and diabetic nephropathy. Metab Clin Exp..

[7] Kikkawa R, Umemura K, Haneda M, Arimura T, Ebata K, Shigeta Y (1987). Evidence for existence of polyol pathway in cultured rat mesangial cells. Diabetes..

[8] Lewko B, Latawiec E, Maryn A, Barczynska A, Pikula M, Zielinski M (2011). Osmolarity and glucose differentially regulate aldose reductase activity in cultured mouse podocytes. Exp Diabetes Res..

[9] Kasajima H, Yamagishi S, Sugai S, Yagihashi N, Yagihashi S (2001). Enhanced in situ expression of aldose reductase in peripheral nerve and renal glomeruli in diabetic patients. Virchows Arch..

[10] Hasegawa G, Obayashi H, Kitamura A, Hashimoto M, Shigeta H, Nakamura N (1999). Increased levels of aldose reductase in peripheral mononuclear cells from type 2 diabetic patients with microangiopathy. Diabetes Res Clin Pract..

[11] Kumar H, Shah A, Sobhia ME (2012). Novel insights into the structural requirements for the design of selective and specific aldose reductase inhibitors. J Mol Model..

[12] So WY, Wang Y, Ng MC, Yang X, Ma RC, Lam V (2008). Aldose reductase genotypes and cardiorenal complications: an 8-year prospective analysis of 1,074 type 2 diabetic patients. Diabetes Care..

[13] Wolford JK, Yeatts KA, Red Eagle AR, Nelson RG, Knowler WC, Hanson RL (2006). Variants in the gene encoding aldose reductase (AKR1B1) and diabetic nephropathy in American Indians. Diabet Med..

[14] Gosek K, Moczulski D, Zukowska-Szczechowska E, Grzeszczak W (2005). C-106 T polymorphism in promoter of aldose reductase gene is a risk factor for diabetic nephropathy in type 2 diabetes patients with poor glycaemic control. Nephron Exp Nephrol..

[15] Sivenius K, Niskanen L, Voutilainen-Kaunisto R, Laakso M, Uusitupa M (2004). Aldose reductase gene polymorphisms and susceptibility to microvascular complications in type 2 diabetes. Diabet Med..

[16] Makiishi T, Araki S, Koya D, Maeda S, Kashiwagi A, Haneda M (2003). C-106 T polymorphism of AKR1B1 is associated with diabetic nephropathy and erythrocyte aldose reductase content in Japanese subjects with type 2 diabetes mellitus. Am J Kidney Dis..

[17] Wang Y, Ng MC, Lee SC, So WY, Tong PC, Cockram CS (2003). Phenotypic heterogeneity and associations of two aldose reductase gene polymorphisms with nephropathy and retinopathy in type 2 diabetes. Diabetes Care..

[18] Fanelli A, Hadjadj S, Gallois Y, Fumeron F, Betoule D, Grandchamp B (2002). et al [Polymorphism of aldose reductase gene and susceptibility to retinopathy and nephropathy in Caucasians with type 1 diabetes]. Arch Mal Coeur Vaiss.

[19] Neamat-Allah M, Feeney SA, Savage DA, Maxwell AP, Hanson RL, Knowler WC (2001). Analysis of the association between diabetic nephropathy and polymorphisms in the aldose reductase gene in type 1 and type 2 diabetes mellitus. Diabet Med..

[20] Moczulski DK, Scott L, Antonellis A, Rogus JJ, Rich SS, Warram JH (2000). Aldose reductase gene polymorphisms and susceptibility to diabetic nephropathy in Type 1 diabetes mellitus. Diabet Med..

[21] Vandenberghe I, Creancier L, Vispe S, Annereau JP, Barret JM, Pouny I (2008). Physalin B, a novel inhibitor of the ubiquitin-proteasome pathway, triggers NOXA-associated apoptosis. Biochem Pharmacol..

[22] Lau J, Ioannidis JP, Schmid CH (1997). Quantitative synthesis in systematic reviews. Ann Intern Med..

[23] Zintzaras E, Ioannidis JP (2005). HEGESMA: genome search meta-analysis and heterogeneity testing. Bioinformatics..

[24] Higgins JP, Thompson SG (2002). Quantifying heterogeneity in a meta-analysis. Stat Med..

[25] Cho WK, Jung MH, Park SH, Baek IC, Choi HB, Kim TG (2012). Association of MICA alleles with autoimmune thyroid disease in Korean children. Int J Endocrinol..

[26] Daghestani MH, Warsy A, Daghestani MH, Al-Odaib AN, Eldali A, Al-Eisa NA (2012). Arginine 16 glycine polymorphism in beta2-adrenergic receptor gene is associated with obesity, hyperlipidemia, hyperleptinemia, and insulin resistance in Saudis. Int J Endocrinol..

[27] Ioannidis JP, Ntzani EE, Trikalinos TA (2004). ‘Racial’ differences in genetic effects for complex diseases. Nat Genet..

[28] Jorde LB, Wooding SP (2004). Genetic variation, classification and ‘race’. Nat Genet..

[29] Yang B, Millward A, Demaine A (2003). Functional differences between the susceptibility Z-2/C-106 and protective Z + 2/T-106 promoter region polymorphisms of the aldose reductase gene may account for the association with diabetic microvascular complications. Biochim Biophys Acta..

[30] McDonough CW, Palmer ND, Hicks PJ, Roh BH, An SS, Cooke JN (2011). A genome-wide association study for diabetic nephropathy genes in African Americans. Kidney Int..

[31] Shimazaki A, Kawamura Y, Kanazawa A, Sekine A, Saito S, Tsunoda T (2005). Genetic variations in the gene encoding ELMO1 are associated with susceptibility to diabetic nephropathy. Diabetes..

